# *Prototheca* Infections and Ecology from a One Health Perspective

**DOI:** 10.3390/microorganisms10050938

**Published:** 2022-04-29

**Authors:** Balázs Libisch, Carine Picot, Andrés Ceballos-Garzon, Monika Moravkova, Marcela Klimesová, Gábor Telkes, Shih-Te Chuang, Patrice Le Pape

**Affiliations:** 1 Institute of Genetics and Biotechnology, Hungarian University of Agriculture and Life Sciences, 2100 Gödöllő, Hungary; 2 EA 1155 IICiMed—Cibles et Médicaments des Infections et du Cancer, Université de Nantes, F-44000 Nantes, France; carine.picot@univ-nantes.fr (C.P.); c-ceballos@javeriana.edu.co (A.C.-G.); 3 Veterinary Research Institute, 62100 Brno, Czech Republic; moravkova@vri.cz; 4 Dairy Research Institute, 160 00 Prague, Czech Republic; marcela.vyletelova@seznam.cz; 5 Department of Surgery, Transplantation and Gastroenterology, Semmelweis University, 1082 Budapest, Hungary; telkesdr@gmail.com; 6 Department of Veterinary Medicine, National Chung Hsing University, Taichung City 402, Taiwan; stchuang@dragon.nchu.edu.tw

**Keywords:** *Prototheca*, protothecosis, mastitis, ecology, food safety, One Health

## Abstract

*Prototheca* microalgae were only recognized as pathogens of both humans and animals in the 1960s; however, since then, these microbes have been drawing increasing interest in both human and veterinary medicine. The first human outbreak of protothecosis in a tertiary care chemotherapy ward in 2018 further highlighted the need to understand in more depth and detail their ecology, etiology, pathogenesis and routes of transmission between different hosts, environments and habitats from a One Health perspective. Protothecal infections have been reported in a growing number of cattle herds around the world in recent decades, and *Prototheca* has become an important bovine mastitis pathogen in certain countries and regions. The survival of *Prototheca* in the environment and its ability to spread in the herd pose a serious challenge to the management of infected dairy farms. Prevention of the disease is particularly important, as there is no effective and reliable treatment for it and the chances of self-healing are minimal. Therefore, the development of more effective drugs is needed for the treatment of human and animal protothecosis. The prudent use of antibiotics and their replacement by alternative or preventive measures, when possible, may further contribute to the control of protothecal infections.

## 1. Introduction

*Prototheca* spp. are unicellular, achlorophyllous microalgae that occur in a wide range of natural habitats, occupying mostly aqueous environments with high organic matter content. *Prototheca* spp. are opportunistic pathogens of both humans and animals, where the precise pathogenic mechanisms of protothecal infections are still mainly unclear. Several *Prototheca* species, namely *P. ciferrii* (formerly *P. zopfii* genotype 1), *P. bovis* (formerly *P. zopfii* genotype 2), *P. wickerhamii*, *P. blaschkeae*, *P. cutis* and *P. miyajii* cause infections both in humans and in animals [1,2]. These observations highlight the importance and validity of One Health principles for current and future studies of *Prototheca* microalgae. Bovine mastitis is the predominant form of protothecal infections in animals; however, protothecosis has also been described in dogs, cats and goats, and sporadic cases have been reported in a number of other vertebrates [3]. 

Although protothecal disease is still considered uncommon, an increasing number of cases are identified globally, and *Prototheca* has gained a growing interest in human and veterinary medicine. *Prototheca* microalgae were first studied in 1894 by Krüger, who identified them as fungi on account of their cultural similarity to yeasts. The pathogenic potential of *Prototheca* at that time was not known [4]. The first animal and human infections by *Prototheca* were reported in 1952 [5] and in 1964 [6,7], respectively. *Prototheca* is now recognized in several countries or geographical regions as a major pathogen of bovine mastitis, and its growing importance was also highlighted by the first human outbreak of protothecosis in a tertiary care chemotherapy ward in 2018 [8,9,10].

The One Health principle recognizes relationships between the health of humans, animals and the ecosystem and encourages interdisciplinary approaches to address complex health issues [11]. *Prototheca* microalgae are adapted to natural environments, such as slime flux, and to man-made environments such as municipal sewage digesters that contain suitable nutrients. From these and from other environmental sources, *Prototheca* microalgae may disseminate into aquatic systems, from where they may enter and pass through the human and animal gastrointestinal tract and enter sewage. Under certain circumstances, such as by traumatic or other types of inoculation, these microalgae can cause opportunistic infections in humans and in domestic or wild animals [12].

The One Health approach can thus be indeed applied to better understand the relatively unexamined and unexplored characteristics of these opportunistic pathogenic microalgae. An analysis of available data concerning their occurrence in various habitats and environments and their role as causative agents of human and animal infections may identify current knowledge gaps regarding their epidemiology and routes of dissemination. Such an analysis could clearly benefit from applying the One Health approach to address one of today’s emerging health challenges.

## 2. The *Prototheca* Genus

Microalgae from the genus *Prototheca* are closely related to the green algae *Chlorella* spp., and both genera are placed within the family *Chlorellaceae,* order *Chlorellales,* class *Trebouxiophyceae*. However, *Prototheca* has lost its ability to synthesize chlorophyll and adopted a heterotrophic nutrition [10,13]. *Prototheca* species are distinguished from other algae, such as *Chlorella*, by their lack of chloroplasts and the presence of a two-layered, instead of a three-layered, cell wall [14]. Comparative analyses of rRNA sequences from various organisms revealed that *Prototheca* has closer relationships with plants than with fungi [15].

The most important animal protothecosis, bovine mastitis, is predominantly caused by *P. bovis*, while *P. wickerhamii* is the most common human pathogenic species [4,6]. Two other species, *P. stagnora* and *P. moriformis*, are commonly isolated from natural environments and are generally considered non-pathogenic. These two species can produce capsule, while this feature is not characteristic for clinical isolates of *P. wickerhamii* and *P. bovis* [1,4,6,16].

Initially, the rRNA gene cluster was the main target for *Prototheca* molecular taxonomic studies. The recently proposed use of the *cytb* gene offered advantages over the previously used rDNA markers because of its high discriminatory capacity, intra-strain sequence homogeneity and technical feasibility, including a reproducible amplification assay [1]. By applying *cytb* gene analyses, genotypes 1 and 2 of *P. zopfii* were elevated to species status in the forms of *P. ciferrii* and *P. bovis*, respectively. Furthermore, five new species were proposed: *P. cerasi*, *P. cookei*, *P. pringsheimii*, *P. xanthoriae* and the re-defined *P. zopfii*. In addition, a new species, *P. paracutis*, has also been recently described [17]. Of the 15 *Prototheca* species, only six (*P. blaschkeae,* *P. bovis,* *P. cutis,* *P. miyajii,* *P. ciferrii* and *P. wickerhamii*) have been clearly implicated as human and animal pathogens [1,8].

**Figure 1:**
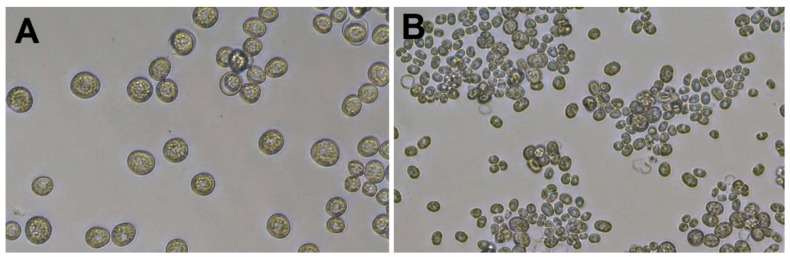
Cells of *P. blaschkeae* (**A**) and *P. bovis* (**B**) microalgae strains grown in RPMI 1640 medium. Images were taken at 200× magnification using a Leica DM IL LED Microscope (Leica Microsystems CMS GmbH, Wetzlar, Germany).

*Prototheca* cells (Figure 1) are non-motile and are much larger than bacteria. As an example, *P. bovis* can be up to 25 μm in diameter [1,6]. The *Prototheca* cell wall does not contain cellulose and chitin, which are constituents of plant and fungal cell walls, respectively. *Prototheca* have a characteristic cell wall of two layers: a thin outer layer and a translucent and thicker inner layer. The membrane-bound plastids of *Prototheca* cells may contain starch deposits and were proposed to represent vestigial chloroplasts of green algae [15].

*Prototheca* cells undergo asexual reproduction through endospore formation subsequent to cytoplasm cleavage. About 2–20 endospores are formed, whose shape, number and size vary among *Prototheca* species. The sporangia (mother cells) break up under pressure from the enlarging spores, and the release of the spores takes place about every 5–6 h in the presence of adequate nutrients [10,18,19]. The sporangia of *P. wickerhamii* (of 3–10 μm size) are generally smaller than the sporangia of *P. bovis* (with a globose to ellipsoidal shape, on average 15 × 13 μm and 9 × 7 μm, respectively) [1,14]. *P. wickerhamii* tends to form symmetrical morula-like shaped sporangia (with endospores arranged symmetrically like a daisy), while *P. bovis* displays more random internal segmentation [20]. In Gram-stained preparations, Gram-positive spores and Gram-negative empty sporangia can be detected [4].

The number of daughter cells increases with the growth rate. The different growth rates also determine particular physiological states that are characterized by a specific cell size and relative DNA, RNA and proteins contents [15,16]. A thick cell wall and no cell divisions are characteristic of a resting cell stage.

**Figure 2:**
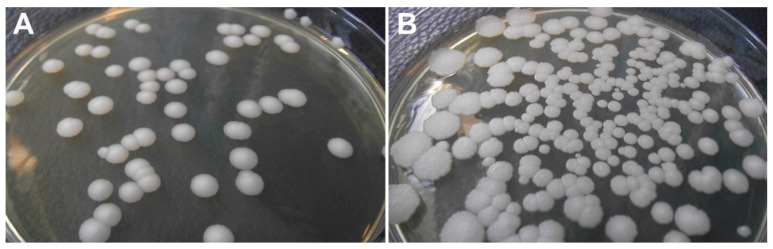
Colonies of *P. blaschkeae* (**A**) and *P. bovis* (**B**) microalgae strains grown on Sabouraud agar medium.

*Prototheca* cells settle rapidly in aqueous environments because of their sufficiently higher density compared to bacteria. Laboratory suspensions and likely the natural populations of *Prototheca* are also hydrophobic, contributing to their affinity for sediments and surface films [12]. Culturing at 30 °C for 72 h is appropriate for most *Prototheca* species, and the mastitis-causing *Prototheca* strains can be cultured at about 35 °C. However, certain strains show a preference for 25 °C, and some slow-growing isolates may require up to 7 days for the formation of visible colonies [6,21]. Culture media containing glucose (such as Sabouraud’s dextrose agar, Figure 2) are suitable for growing *Prototheca*, where 100 μg/mL chloramphenicol can be added to suppress bacterial growth [16]. *Prototheca* microalgae may not survive freezing for longer periods of time, and fresh samples are therefore recommended for culturing in situations when a culture-based analysis is required [22].

## 3. *Prototheca* and Bovine Mastitis

*P. zopfii* was initially identified as the causative agent of bovine mastitis in Germany in 1952 [5,13]. Mastitis caused by *Prototheca* species represents the most important form of protothecosis in animals because of the associated economic losses (reduced milk production, premature culling of affected animals and veterinary expenses) [23,24].

Protothecal mastitis has been reported worldwide, including in the USA, Canada, Belgium, Denmark, Germany, Italy, Poland, Hungary, Brazil, Taiwan, Korea, China, Japan and in other countries [16,23]. It was proposed that endemic *Prototheca* mastitis with high numbers of affected cows could be identified mainly from geographical regions with a relatively high humidity and/or temperature, supporting the increased growth of these microalgae in the environment [25]. During a *P. zopfii* outbreak of clinical bovine mastitis in the State of São Paulo, Brazil, 77/211 (36.49%) milk samples proved positive for *P. zopfii*, as well as fecal samples of 11 calves and two cows and two swabs from teat cup rubbers. The microalgae could be also isolated from two water samples and one soil sample collected from the dry cow pasture [26]. A similar level of prevalence for *P. zopfii* (34.1%, in 14/41 lactating cows) was found during an outbreak of protothecal mastitis in Taiwan [27]. In Canada, the herd-level prevalence of *Prototheca* spp. was found to be around 6% based on bulk tank milk examination using a commercial polymerase chain reaction (PCR) assay [28]; in Poland, *P. bovis* was isolated from quarter milk samples on eight farms out of 16 examined [29].

Protothecal mastitis is usually recognized as a chronic and asymptomatic disease which causes increased somatic cell counts and reduced milk production due to the damage inflicted on the udder [30]. The clinical form appears much less frequently and is characterized by milk alterations (watery appearance and the presence of white flakes) and changes in the udder (swelling, hard tissue consistency, tenderness). However, *Prototheca* spp. as a causative agent of mastitis are often neglected, and protothecal mastitis is often suspected when resistance to antibiotic therapy is observed [31]. Out of the *Prototheca* spp., *P. bovis* is the most common cause of clinical or subclinical mastitis in cattle. Sporadically, *P. blaschkeae* has been documented [32]. *P. wickerhamii,* the main causative agent in human infections, has rarely been described in mastitis milk [33].

During the development of bovine mastitis, *Prototheca* cells probably enter the udder through the teat orifice, originating, for example, from contaminated milking equipment or from various environmental sources [34]. The reported level of infection within dairy herds is variable. In some herds, only a few animals are infected, but in other herds, *P. bovis* was detected in up to 30% of mastitis milk samples or more [35]. The mean prevalence in a herd is usually about 10% [29,36]. Although it is a persistent infection, unfortunately it is not always possible to detect all infected individuals in the herd because of intermittent shedding. Therefore, the re-examination of suspected cows is recommended to reveal all infected cows in the herd [37].

The infection is usually confined to the mammary gland and regional lymph nodes exhibiting granulomatous inflammation [4,23]. *P. bovis* may survive in the mammary gland during the dry period and be released back into the milk during subsequent lactation. However, in addition to the excretion of *Prototheca* spp. in milk, *Prototheca* spp. are also spread into the environment through feces [38]. Calves fed with *Prototheca*-containing mastitic milk may be a further source of environmental contamination on impacted farms [16]. *Prototheca* can be also isolated from various specimens (feces, mouth, nose, rectum and vagina) from cows whose milk was negative for *Prototheca* spp., suggesting that *Prototheca* spp. can also colonize the bovine gastrointestinal and urogenital tract without causing harm to the host [23]. Milk samples from 25 cows with mastitis and 24 samples of bulk-tank milk were examined for the presence of *Prototheca* in a study conducted in Japan [39], together with 360 specimens from cow-barn surroundings (drinking water, sewage, feces and sawdust). All 67 mastitis isolates were identified as *P. bovis* (i.e., *P. zopfii* genotype 2). However, among 32 isolates recovered from cow-barn surroundings only three isolates were identified as *P. bovis*, while the other 29 isolates were *P. ciferrii*, indicating that both *Prototheca* species could exist in the cow-barn surroundings, but no sites were frequent sources of *P. bovis*. It was therefore presumed that the route of dissemination for *P. bovis* was probably through milking equipment contaminated by protothecal mastitis [39].

Analyses of risk factors for *Prototheca* mastitis on dairy farms in Ontario, Canada indicated that the off-label intramammary administration of injectable drugs, antimicrobial treatment and multiple and possibly unsanitary intramammary infusions were associated with an increased risk of *Prototheca* mastitis [40]. The results of a cross-country study performed in Poland suggested that the size of the farm (in acreage) and the number of farm employees positively correlated with the incidence of *Prototheca* mastitis [29]. A study examining five dairy herds in the Apulia Region in Italy indicated that the presence of *Prototheca* spp. in dairy herds seemed to correlate with the hygienic conditions of the milking equipment and with the proper management of animal feeding and drinking water [41].

## 4. Protothecosis in Other Animals

Besides bovine mastitis, protothecosis has also been reported as an uncommon disease in a number of other animal species, including dogs (*Canis lupus familiaris*), cats (*Felis catus*), roe deer (*Capreolus capreolus*), goats (*Capra hircus*), horses (*Equus caballus*), a beaver (*Castor canadensis*), a cape hyrax (*Procavia capensis*), Atlantic salmon (*Salmo salar*), carp (*Cyprinus carpio*), fruit bat (*Rousettus lanosus*), a flying fox (*Pteropus lylei*) and a corn snake (*Elaphe guttata guttata*) [2,42,43,44,45,46].

While mastitis is the predominant form of protothecal infection in cattle, dogs typically suffer from disseminated infections where the outcome is usually fatal. Until recently, 59 infected dogs had been reported worldwide [8,47]. Dogs acquire *Prototheca* after the ingestion of large numbers of microalgae [8]. Therefore, infections often begin with chronic bloody diarrhea, followed by neurologic symptoms such as ataxia, blindness, deafness or seizure, with *P. zopfii* genotype 2 (that is, *P. bovis*) being most frequently isolated from canine protothecosis and, in rare cases, *P. wickerhamii* [4,48].

Protothecosis in cats has rarely been reported and usually causes nodular skin lesions in sites of penetrating injury, for example on the face, head or the distal limbs. The predominant species isolated from feline protothecosis is *P. wickerhamii* [8,49,50], while Huth et al. [51] reported a case of *P. bovis*-induced inflammation of the nasal skin and cutaneous mucosa of the right nostril in a 14-year-old cat. Protothecal infections have also been described in goats with nasal and cutaneous forms caused by *P. wickerhamii* [43]. Protothecosis of a fruit bat resulted in lesions in the lymphatic system, spleen, the central nervous system, heart, muscle and kidneys, causing severe granulomatous lymphadenitis and splenitis and a widespread granulomatous meningoencephalitis [42]. *P. wickerhamii* was isolated from a carp (*Cyprinus carpio*) which was underweight for its age, sluggish and displayed erratic swimming behavior (ataxia). Skin erosions and ulcerative and nodular lesions were spread over its whole-body surface. Similar lesions could be observed on several of its internal organs, including the liver and the intestinal mucosa [2].

During the course of a mouse protothecal mastitis model experiment [52], 6–8 weeks old lactating female mice were inoculated intramammarily with either *P. bovis* (50 µL containing 1 × 10^5^ CFU/mL) or an equal volume of phosphate buffered saline (the control group). *P. bovis* induced acute mastitis with the infiltration of leukocytes throughout the parenchyma and in the lumen of alveoli. *P. bovis* cells were present both free within alveolar lumen and throughout the interstitium of the mammary tissue. Macrophages were found in the mammary interstitium and neutrophils were diffusely distributed in *P. bovis*-infected mice. The presence of *P. bovis* upregulated gene activity and protein production of pro-inflammatory TNF-α, IL-1β and Cxcl-1 in the mammary tissue at four days post inoculation [52]. *P. bovis* caused model mastitis in mice that manifested in the severe red swelling of the mice mammary glands as well as necrosis and nodules lesions in the infected mice mammary tissue, accompanied by macrophage and neutrophil infiltration [53].

In addition to the various types of infections caused by *Prototheca*, several reports also described the asymptomatic colonization of animals by the microalgae. As an example, 11/15 pig and 3/11 dog feces samples, respectively, examined in the Philippines were colonized by *Prototheca*, predominately *P. zopfii*, while other sampled animals proved negative [12]. *P. wickerhamii* was also recovered from a pigeon crop sample, but not from cloaca or droppings. It was proposed that the isolation of *P. wickerhamii* from the pigeon crop might be explained by the pigeon swallowing contaminated water [54]. *P. zopfii* was detected in 3/14 (21.4%) fecal samples of wild boars by using selective *Prototheca* isolation medium (PIM) [21,55]. Fecal samples of rats trapped on a small rural pig farm tested positive for *P. zopfii*, where contaminated feed may have been the source of the algae and the animals did not sustain their intestinal colonization when their feed was *P. zopfii* free [56]. *P. zopfii* was also isolated in monoculture from 9/146 (6.2%) horse fecal samples [57], indicating that a range of wild and domestic animals may harbour *Prototheca* in their gastrointestinal tract and disseminate *Prototheca* spp.

## 5. *Prototheca* in Natural, Agricultural and Other Human-Impacted Environments

Only few targeted environmental studies have been performed for *Prototheca*, and in several cases these investigated the environments of farm animals. Our understanding of the ecology of *Prototheca* microalgae is therefore rather limited. Species formerly designated as *P*. *zopfii* (either *P*. *bovis* or *P. ciferrii*) tended to be the most abundant in the environments of cattle farms [34]. Watering troughs, manure, feed and mud were *Prototheca*-positive environmental samples at agricultural farms in one study [23], where drinking water and manure were the major environmental reservoirs, similar to cases before. Wet feeds rich in starch and oligosaccharides, such as potato pulp, may also serve as a medium for *Prototheca*. The microalgae have been detected in milking equipment, in its pipelines, on teat cup rubbers and may even survive routine disinfection procedures with chlorine solution [16]. In a cross-country study conducted in Poland, bedding was the most *Prototheca*-abundant sample type among environmental sources, followed by barn walls, feed and drinking water. Environmental samples were most commonly positive for *P. bovis* (47.6%), followed by *P. ciferrii* (33.3%) and *P. blaschkeae* (19.1%) [29]. *P. blaschkeae* was also cultured from fecal and environmental samples of pig farms [58]. Furthermore, *P. zopfii* and *P. wickerhamii* were detected in freshwater aquariums and aquarium filters [12].

In terms of natural environments, the slime flux of certain deciduous trees is a habitat for *Prototheca* spp., such as in elm trees (*Ulmus americana, U. carpinifolia*)*,* Japanese elm (*Zelkova serrata*), lime trees (*Tilia* spp.)*,* Mizunara (*Quercus crispula*) and white mulberry (*Morus alba*)*. P. stagnora* was found to be a habitant of older harvested banana (*Musa sapientum*) and plantain (*M. paradisiaea*) stumps, while *P. wickerhamii* colonized fresh *Musa* sp. stumps and the flower bract water of *Heliconia* sp. [59]. *P. zopfii* was also isolated from broccoli leaves [60], and two *P. paracutis* strains were isolated from the water and soil of a mangrove forest in Thailand [17]. *Prototheca* microalgae are not known as pathogens of plants. It is possible, however, that they play a certain role in slime flux formation, probably together with other microbes. *Prototheca* spp. were also present in soil specimens from beneath trees producing slime flux and from stream banks and in pasture soil [12].

The microalgae were cultured from sewage of different geographical locations, including Ohio, France, Spain and the Philippines. *P. zopfii* and *P. wickerhamii* were isolated from raw sewage and sludge from both aerobic and anaerobic wastewater treatment plants. *P*. *wickerhamii* is likely the most abundant species in human sewage [34], and it was suggested that the primary and secondary sewer system is a place of growth and reproduction for *Prototheca*. *Prototheca* spp. are generally killed by chlorination in the liquid effluent from settling ponds, but in certain cases of inadequate treatment they may escape into rivers [12]. These earlier observations led us to initiate a pilot bioinformatic analysis of shotgun metagenomic sequencing data for wastewater samples available in the National Center for Biotechnology Information Sequence Read Archive (NCBI SRA) database. The initial results detected *P. zopfii*/*P. bovis* microalgae in two wastewater metagenomic datasets from the USA and Pakistan, respectively (Libisch et al., unpublished data, Supplementary Table S1), indicating the potential utility of applying metagenomic methods to gain further insights into the ecology of *Prototheca* spp.

Concerning occurrences in food, Bacova and colleagues [61] monitored *Prototheca* spp. in milk samples obtained from supermarkets in the Czech Republic from different producers. *P. bovis* was found in 13 out of 16 milk samples at concentrations ranging from 1.50 × 10^0^ gene copies/mL up to 1.18 × 10^4^ gene copies/mL using real-time PCR. However, all samples were culture negative, confirming pasteurization effectivity in these milk samples [61]. On the other hand, in a study performed in Brazil *Prototheca* was detected by plate count in a cheese sample produced using *Prototheca*-contaminated milk [62]. The *Prototheca* spp. counts in bulk milk samples ranged from 1–3 × 10^4^ colony forming units/mL (CFU/mL), while the examined cheese sample produced from contaminated milk contained *Prototheca* spp. at 7.5 × 10 CFU/mL [62]. In another study, raw milk and locally manufactured cheese samples were collected and analyzed from city markets in Qena Governorate, Egypt [63]. *P. bovis* was detected by PCR and had the highest incidence among *Prototheca* spp. in both the examined raw milk and cheese samples. The isolation of *Prototheca* from the examined cheese samples might have been due to the use of raw milk for cheese production, simple processing methods or alternatively contamination after heat treatment or the during production and handling steps of the cheese [63].

*Prototheca* microalgae were also found in other food products, such as in beef, pork, clams and crabs. Although there is a variety of sources from which they can be isolated, sewage water and animal waste probably represent their main environmental reservoir [15]. In a study conducted in China in Hubei province, a warm and humid region, *P. bovis* was found in brewer’s grains (an important feed additive for dairy cattle in China) and in fresh cow feces from a dairy farm affected by *P. bovis* infections. Moreover, *P. bovis* was also isolated from the truck transporting the brewer’s grains to the dairy farm, suggesting that such vehicles may also possibly disseminate *P. bovis* [53].

## 6. *Prototheca* in Human Disease

*P. wickerhamii*, the most common human pathogenic species, was described as a new species by Japanese scientists in 1959 [6]. Davies and colleagues published the first description of a human infection attributed to *Prototheca*, which concerned a 31-year-old rice farmer from Sierra Leone, in 1964 [7]. The patient had a papular, hypopigmented small lesion on his right foot that had gradually encircled the foot and spread up the leg. The patient was lost to follow-up after 1965 and presumably died with or because of his infection [7,14].

Human protothecosis has been classified in three main clinical forms, namely cutaneous lesions, olecranon bursitis and disseminated or systemic infections [20,64], and most infections are probably caused by a traumatic inoculation into subcutaneous tissues. In an analysis of 160 cases of human protothecal infections published before June 2011, more than half of the cases (93/160, 58.1%) were of the cutaneous form [6]. The incubation period for protothecal infections is not well documented; however, periods of weeks to months were suggested. A local or systemic immunosuppressive factor was identified in about half of the human cases. *P. bovis* appears to be the main *Prototheca* species in systemic protothecal infections, while *P. wickerhamii* is involved to a greater extent in cutaneous human infections. Workers, for example, in rice paddies, fishermen, farmers, handlers of raw seafood and aquarium staff were considered to have a higher risk of exposure to *Prototheca* species [20].

The incidence of protothecosis was roughly equal among males and females. The patients’ ages ranged from 78 days to 88 years, but it was skewed toward older ages. Skin infections comprised over 50% of the cases, and other infection types included disseminated infections, olecranon bursitis, wounds, septicaemia, nail lesions, peritonitis and miscellaneous other cases. Skin infections had the best prognosis, with a cure or improvement rate of about 78% and a mortality of only 1%, while disseminated cases had the worst prognosis, with about a 33% cure or improvement rate and a 56% mortality rate [6]. Overall, human protothecosis appears to be a rare disease with an increasing incidence in recent decades, with the increase being mostly attributed to patients with immunosuppression, corticosteroid treatment or both [6]. Hospital-acquired cases were mostly associated with surgery or orthopaedic procedures. Infections may occur when skin injuries come into contact with microalgae-contaminated water, while colonized patients with predisposing factors may develop endogenous protothecal infections [14].

The carriage of *Prototheca* in the human gastrointestinal tract has also been described, although in an earlier study it occurred sparingly in human feces (three positives out of 300 samples) [65]. In another report, a 2-month-old infant diagnosed with treatment-resistant gastroenteritis provided *P. wickerhamii*-positive stool cultures in the context of an otherwise usual intestinal flora [66]. In a recent survey in a rural area in Thailand [67], *P. bovis* was detected in the fecal samples of four out of 98 healthy volunteers exhibiting no diarrhea. Participants of this study spent extended periods of time on rice fields (similar to the first human case described by Davies [7]), and had frequent contact with ruminants and poultry, whose manure was used to fertilize local vegetable gardens. Since it was not possible to confidently identify the source of protothecal colonization, the authors highlighted the need to apply a comprehensive One Health approach that includes humans, animals and the environment in such future surveys [67].

## 7. Diagnosis and Treatment of *Prototheca* Infections

*Prototheca* infections can be diagnosed by histopathological examination and/or by the isolation of the causative agent [20]. Traditionally, *Prototheca* isolates were identified by their macro- and micromorphology and biochemical profiling. The creamy-white colonies can be mistaken for yeasts; however, *Prototheca* endospores are characteristic features for identification by microscopy [4,15]. Matrix-assisted laser desorption/ionization-time of flight (MALDI-TOF) mass spectrometry proteomic analysis [6,10,58] and various molecular methods have also been developed for the identification and detection of *Prototheca* spp. [9,34,35,36,39,51,58,61]. Furthermore, enzyme-linked immunosorbent assay (ELISA) may be applied for discrimination between infected and non-infected dairy cows, and the detection of anti-protothecal antibodies in serum and whey provided sufficient specificity and sensitivity for the diagnosis of protothecal mastitis [9,66].

Definitive treatment guidelines for protothecal infections have not yet been established. This is because the algae often display high levels of resistance to a variety of antimicrobial agents and there is a lower correlation between in vivo clinical response and in vitro susceptibility results [20]. This low correlation is probably linked to the absence of guidelines for in vitro susceptibility testing for *Prototheca* microalgae and to the absence of established clinical breakpoints. Many aspects of in vitro susceptibility testing of *Prototheca* species are similar (or even identical) to those of the Clinical and Laboratory Standards Institute (CLSI) or European Committee on Antimicrobial Susceptibility Testing (EUCAST) procedures recommended for yeasts. However, specific conditions for protothecal growth should be considered when optimizing antimicrobial susceptibility testing for these organisms in future [20,68].

Standard treatment guidelines have not yet been established for human or for veterinary protothecal infections [69,70]. *Prototheca* spp. share some similar features with yeasts, specifically the presence of ergosterol in the cell membrane. For this reason, treatment of human protothecosis most commonly includes antifungal agents plus surgical approaches [20]. The surgical treatment should constitute complete excision because drainage might fail, provided the human protothecosis was proven before the intervention [71]. Wide surgical excision has been indicated for protothecal cutaneous lesions of dogs and cats, although a substantial number of animals subjected to excisional procedure or biopsy of solitary nodules developed systemic infection following the surgery [72].

Intravenous amphotericin B is currently considered the most effective treatment for human protothecal infections, with a cure or improvement rate of 72%. Itraconazole and fluconazole had cure or improvement rates of 71% and 65%, respectively. For relatively mild cases, it was recommended to begin with oral itraconazole or fluconazole and to use intravenous amphotericin B for serious infections and for infections that have failed azole treatment [6]. In addition, in vitro testing has shown that ravuconazole has a higher algaecide effect than other azoles tested against *Prototheca* species. This new azole agent, available since 2018 in Japan, may also be considered for the treatment of human and animal protothecosis [8].

In general, *Prototheca* spp. tested resistant to 5-flucytosine, and *P. wickerhamii* and *P. bovis* isolates showed variable resistance against fluconazole and itraconazole or voriconazole. Currently, little information is available about the resistance mechanisms responsible for the observed species- or strain-specific in vitro antimicrobial susceptibility patterns of *Prototheca* algae. Most polysaccharides in the *Prototheca* cell wall are β-1, 4-bonded rather than β-1, 3; thus, the echinocandins which inhibit β-1, 3 glucan synthesis are generally ineffective [8].

In the course of a chronic human meningitis case caused by *P. wickerhamii* in Japan, during a 3-year period of therapy, the induction of secondary drug resistance was reported. The minimum inhibitory concentrations (MICs) for amphotericin B and fluconazole increased from 0.39 to 3.13 μg/mL and from 50 to 200 μg/mL, respectively. This extent of clinically significant acquired resistance was regarded to be an uncommon phenomenon [73]. To date, no effective therapies against protothecal mastitis have been developed or routinely applied. Liposomal amphotericin B (with reduced nephrotoxicity, applied in human therapy) is highly expensive and thus unsuitable for routine use in cattle farming. Furthermore, in the European Union (EU), while amphotericin B is authorized in human medicines, there are no such authorized veterinary medicines in the EU. Nonetheless, textbooks suggest that amphotericin B has been used to treat systemic fungal infections in companion animals and certain fungal diseases in horses [74]. Amphotericin B cannot be used in food-producing animals in the EU, as it is not included in the Annex to Regulation (EU) No 37/2010 on pharmacologically active substances and their classification regarding maximum residue limits in foodstuffs of animal origin [74].

When protothecal infection is detected in a cow, the elimination of the animal from the herd is usually necessary. To avoid problems associated with protothecosis, the farmers are advised to follow preventive measurements against protothecosis. Excessive empirical use of antimicrobials should be avoided whenever possible, and the therapy should be based on the results of the laboratory identification of the causative agent of mastitis and its in vitro antimicrobial susceptibility pattern. Good hygienic conditions with respect to milking equipment should be maintained and the proper management of animal feeding and drinking water is advised [13,39,41]. 

Taken together, the development of novel and more effective agents is needed for the treatment of human and animal protothecosis [10], together with the establishment of standardized procedures for in vitro susceptibility testing. Some recent in vitro studies have shown various promising, novel algicidal treatments, such as silver nanoparticles [75] or guanidine [76], but their in vivo efficacy has not yet been demonstrated [34]. The expanding knowledge based on the sequencing of *Prototheca* genomes may contribute to a better understanding of their pathogenesis and pathways of infection and to developing specific immunisation procedures or algicidal substances against protothecal infections [13].

## 8. Resistance of *Prototheca* spp. against Environmental Conditions

*Prototheca* microalgae are likely protected from digestion in the gastrointestinal tract due to their sporopollenin cell wall. Sporopollenin is a natural organic polymer resistant to mechanical stress, physical treatment, degradation by acidic and alkaline hydrolysis or enzymatic breakdown. Laboratory experiments indicated that *Prototheca* spp. were unharmed by passing through rodent and primate intestinal tracts [6,12,48]. *P. bovis* strains were shown to tolerate pH 2.1 and 6.0% NaCl concentration, while *P. blaschkeae* strains had a lower salt and pH tolerance of pH 4.0 and 4% NaCl, respectively [24].

One of the important mechanisms that enable *Prototheca* spp. to persist in the environment is the formation of biofilms, which contributes to the resistance of *Prototheca* microalgae against various sanitizers on farms. *Prototheca* biofilms also displayed an increased resistance to antimicrobial agents [34,77]. The chlorination of effluents from wastewater treatment was variably effective in reducing the numbers of *Prototheca.* It was proposed that inadequate or no chlorination, heavy organic loading or high effluent volumes may result in the discharge of large numbers of *Prototheca* spp. into rivers and lakes [65].

The susceptibility of *Prototheca* algae to pasteurization has, thus far, only been examined in a small number of studies, with variable results [78,79,80,81]. Forty *P. zopfii* strains isolated from cow milk were subjected to different heat-treatments (72–75 °C for 15 s, 72–75 °C for 20 s and 62–65 °C for 30 min), where the resistance of 34 strains was reported in at least one of the conditions [4,78]. In another study, the effect of eight different temperature/time ratios on *P. bovis* and *P. blaschkeae* isolates was tested in the range of 62 °C for 15 min to 100 °C for 1 s [79]. Total growth inhibition was achieved only by using the 100 °C for 1 s treatment, suggesting that ultrapasteurization was the only procedure capable of ensuring that the milk becomes free of algae. A significant difference was found between the susceptibilities of the two species: *P. blaschkeae* displayed higher resistance to heat treatment than *P. bovis*, with an adjusted logCFU/mL mean count value 1.3-fold higher than that of *P. bovis* [79]. Lassa and colleagues reported that 13/50 (26%) of tested *P. zopfii* isolates demonstrated resistance to 72 °C for 15 s treatment, providing a concentration of 5–29 CFU/mL of milk [80]. Another recent work examined the susceptibility of five *P. bovis* and one *P. blaschkeae* isolates in the temperature range of 47–60 °C for 1–90 min [81]. In this study, all the *Prototheca* strains survived the 50 °C for 90 min treatment, but exposure to 60 °C for one minute was effective. Therefore, it was proposed that the commonly used pasteurization temperature of 72 °C for 15–20 s during the manufacturing of dairy products would eliminate *Prototheca* spp. and would ensure that the health and safe status of the product was not be compromised [81]. This latter finding agrees with the views of other authors [22], suggesting that the human health risk from the consumption of processed milk from sub-clinically infected cows is low, as pasteurization is usually effective against *Prototheca*. On the other hand, the consumption of raw milk from *Prototheca*-infected cows may still pose a potential human health risk [22]. Taken together, the experimental results available in the cited reports are not yet conclusive. One possible explanation for the observed variable heat-treatment susceptibility may be the tendency of these microalgae to form cell clumps, thus preventing adequate exposure of the algal cells in the middle of clumps to elevated temperatures. In this case, the homogenization of the milk during heat treatment may also potentially contribute to a more effective pasteurization process [79].

When *Prototheca* microalgae have been introduced into a farm, their eradication may be laborious. Due to dust or feces contamination on the teat skin before milking and fat and protein contamination after milking, respectively, dipping teats through the use of effective disinfectants before and after milking is recommended to prevent infections by environmental and contagious pathogens. Chlorhexidine and iodine are both suitable for the disinfection of teats and milking equipment under normal circumstances or during outbreaks of protothecal infections [82,83,84,85]. Minimal algaecide concentrations (MAC) of chlorhexidine and povidone-iodine were obtained for 16 *P. zopfii* isolates from Taiwanese dairy farms by the microdilution method, where the best concentrations of chlorhexidine and iodine for in vitro algaecide efficacy were 3.13 μg/mL and 390.63 μg/mL, respectively. These identified disinfectant concentrations were lower than that of the manufacturer’s recommendations [86].

## 9. One Health Perspective

**Figure 3:**
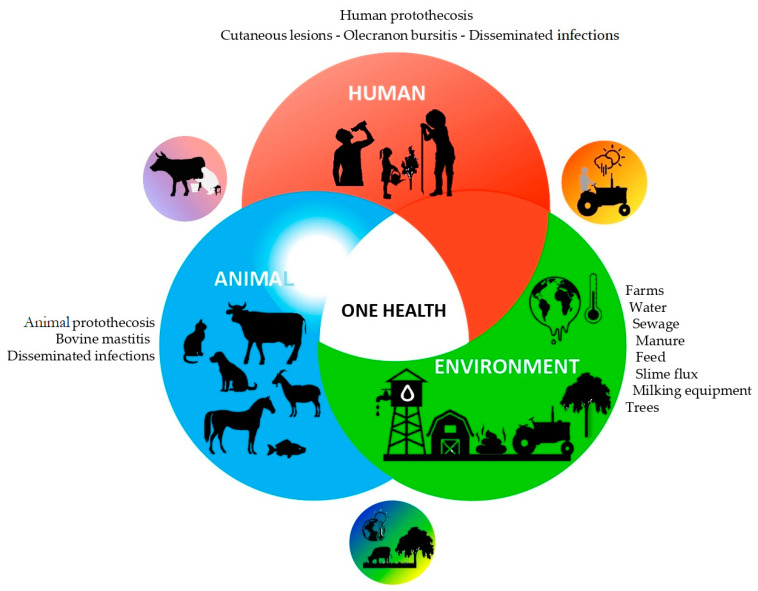
*Prototheca* infections and ecology from a One Health perspective: a schematic diagram summarizing the diverse habitats, human and animal hosts and possible routes of transmission for *Prototheca* microalgae.

The culture-based studies performed by Pore and colleagues to screen for and to isolate *Prototheca* microalgae from a wide range of environmental samples and food products using PIM medium [21] provided highly valuable findings on the ecology of *Prototheca* microalgae and their potential routes of dissemination between different natural and human-impacted environments and habitats (Figure 3) [12,56,59,65]. In these studies, *Prototheca* spp. were also recovered from drinking water and various food products in the Philippines, and *P. wickerhamii* was isolated from the drinking water samples. Salads were contaminated predominately by *P. zopfii,* probably due to the ice used to chill the lettuce. *Prototheca* were detected from pork products, beef and carabao products, clams, crabs and from raw pork sourced in the markets. It was assumed that meat products were probably contaminated by pig intestinal contents because of the applied local abattoir practices. Effluents of slaughterhouse wastes in West Virginia, USA, were also found to be rich in *Prototheca* algae in another study [12]. 

An analysis of protothecal canine infections in Australia found that the cases occurred generally in medium to large breeds, likely living outdoors [47]. It remained unclear exactly how and why the microalgae initiated these infections. However, it was proposed that the first step could have been the colonization of the colon after the ingestion of high numbers of *Prototheca* cells from an environmental source, such as from dam water or stagnant pools. This would explain the observed overrepresentation of larger dogs with usually outdoors domiciles. Presumably, the next step of infection might have involved a disruption of the epithelial barrier of the bowel wall, thus permitting access for *Prototheca* to the submucosa [47]. A portal for internal inoculation may also have been provided by trauma, surgery or possibly through the bites of insects that populate the slime flux of trees [15].

Other observations pointed to the assumption that under certain circumstances *Prototheca* may also disseminate via the air, aerosols or by droplet infection. *P. zopfii* was identified in air samples from semi-closed pig farms evaluated in Korea, suggesting that *Prototheca* spp. may also be airborne [87,88]. In a report describing pyogranulomatous rhinitis and necrotizing sinusitis associated with *P. bovis* infection in two horses [44], it was noted that both the horses lesions developed only in the upper respiratory tract and that the infection therefore likely occurred via inhalation. The source of infections remained uncertain, although in one of the cases the pasture was located adjacent to that of dairy cows, and *P. bovis* is predominant in bovine protothecal mastitis [44]. 

*Prototheca* spp. can colonize the human skin, fingernails, the respiratory tract and digestive system. Infections may develop after the traumatic inoculation of contaminated soil, water or other materials [10,20]. Molecular typing of strains from environmental samples together with those causing the infection could contribute significantly to uncover the etiology of protothecal infections, if they can be closely linked to each other by genotyping. If the environment is the main source of protothecal infections, disease prevention may involve reducing the environmental load by microalgae (for example by chemical treatment of contaminated water), protecting subjects (such as by separating contaminated areas) or reducing pathogen load at the entry site through topical treatment [6].

The detection of *Prototheca* in pigeon crop and rat feces [54,56] point to the possibility of their role as vectors in the spread of microalgae between various environments or groups of animals [56]. However, neither animal-to-human nor human-to-human *Prototheca* transmission has been proven to date [9,15]; thus, to investigate the possible modes of transmission would require further targeted studies in the future.

Milk and dairy products contaminated with *Prototheca* algae have been proposed to be potential sources of human infection or colonization [4,41,79,89,90]. Costa and colleagues reported a relevant observation, when the occurrence of human enteritis was associated with and followed the consumption of cheese contaminated with *Prototheca* sp., and the algae were subsequently isolated from this patient’s feces [56,89,90]. The National Center for Epidemiology in Hungary examined the case of a 10-year-old girl suffering from bloody diarrhea on a rural farm [91]. Negative stool cultures were obtained on selective media for enteric bacterial pathogens, and the patient’s parasitological examination was also negative. However, mycological culture yielded a *Prototheca* sp. strain. It was not established that the bloody diarrhea was actually caused by the *Prototheca* strain, but the circumstances (farm lifestyle) made it conceivable that the consumption of raw milk was a possible source of infection. During repeated tests, her stool culture became *Prototheca*-negative along with the cessation of diarrheal symptoms. No information is available whether any therapy was applied during this case, and, if so, what medication was administered [91]. Similarly, a study of Joerger and colleagues [92] described a case of chronic meningitis due to *P. zopfii* in an adolescent girl who lived on a farm in rural Pennsylvania with her family. The source of *P. zopfii* infection was not identified; however, it was noted by the authors that she not only swam in freshwater ponds, but also had frequent exposure to soil on her family’s farm and frequently milked cows [92].

The consumption of milk and/or dairy products contaminated with *Prototheca* spp. [93] may represent one potential route of transmission. Although direct human infection through dairy products has not been never confirmed, it was confirmed that *Prototheca* spp. could be extracted to milk in relatively high quantities [61]. *Prototheca* cells do not usually survive pasteurization temperatures [61], although in a few studies the survival of *Prototheca* spp. was documented, with the same being true in the case in non-pasteurized cheese [62,63]. These observations support the necessity to implement effective control measures on dairy farms and appropriate quality control practices for milk products [79]. Further studies are therefore needed regarding the effect of common pasteurization regimens on *Prototheca* contaminated dairy products and regarding the potential public health risks associated with the consumption of raw milk from *Prototheca*-infected cows.

The prudent use of antibiotics is one of the key objectives of the One Health principles. In this respect, it is of specific relevance that antimicrobial treatment and multiple and possibly unsanitary intramammary infusions were identified as factors associated with an increased risk of *Prototheca* mastitis [40]. An analysis performed in Germany also suggested that antibiotic treatment could promote protothecal infections through inhibiting the competitive natural udder bacterium flora [16,66,94,95]. Pieper and colleagues recommended that a farmer–veterinarian relationship should be established, and treatment options discussed to avoid excessive, unsuccessful and extra-label antibiotic use for mastitis because *Prototheca* microalgae might act as opportunistic pathogens and may be promoted by antibiotic-induced suppression of the natural udder flora [96]. Consequently, excessive empirical use of antibiotics was also suggested by other authors to be avoided whenever possible, as the administration of antibiotics was considered useless, or even counter-productive, in the case of confirmed protothecal or *Candida* mastitis [13].

The effects of global climate change should also be considered when assessing the medium to long-term pathogenic potential of *Prototheca* microalgae from a One Health perspective. Higher temperatures often combined with high humidity might enhance the multiplication of the microalgae in the environment [4,13]. In Australia, although overall case numbers were low, canine protothecosis was found to be more common in South-East Queensland, a warm sub-tropical region of the continent. Furthermore, no canine protothecosis cases were identified from Tasmania and New Zealand during the study period, in two regions with cooler climates [47]. It was also reported that the occurrence of environmental mastitis may be influenced by the season of the year, with the rate of new infections being highest during the summer and periods of rainy weather, probably due to an increased number of microbes in the environment. In line with these reports, a statistically significant (*p* < 0.05) increase of environmental mastitis was detected during hot and wet weather, that is, in September to February, in a study conducted in Brazil [25].

## 10. Conclusions

*Prototheca* microalgae were recognized as pathogens for both humans and animals only in the 1960s; however, since then, these microbes have been drawing increasing interest in human and veterinary medicine. The first human outbreak of protothecosis, reported in a tertiary care chemotherapy ward in 2018, further highlighted the need to thoroughly understand in detail the ecology, pathogenesis and routes of transmission of *Prototheca* spp. between different hosts, environments and habitats from a One Health perspective. Several *Prototheca* species, namely *P. ciferrii*, *P. bovis*, *P. wickerhamii*, *P. blaschkeae*, *P. cutis* and *P. miyajii*, were shown to cause infections in both humans and animals.

*Prototheca* spp. have been also isolated from a wide range of environmental and other sources, including drinking water, raw milk, certain types of cheese, salads, lettuce and broccoli, raw meat and meat products, farm environments, animal feed, gastrointestinal and fecal samples from domestic and wild animals, a truck transporting cattle feed, farm air samples, sewage and sludge from wastewater treatment plants.

These observations indicate that, in addition to environmental reservoirs, a range of wild and domestic animals may potentially harbor *Prototheca* in their gastrointestinal tract and disseminate *Prototheca* spp. *Prototheca* microalgae can be isolated from milk in relatively high quantities; thus, the consumption of contaminated milk and/or dairy products may also represent a potential route of transmission. The actual source of protothecal infections remained uncertain in a number of cases. Furthermore, neither animal-to-human nor human-to-human *Prototheca* transmission has been proven unambiguously. Therefore, the molecular genotyping of *Prototheca* strains cultured from the environment together with those causing the infection would be necessary in future studies. A comprehensive One Health approach must include concurrent testing of human, animal and environmental samples involving the soil, water, air and aerosols.

The available experimental data regarding the susceptibility of *Prototheca* algae to milk pasteurization are not yet conclusive. Further and larger-scale studies are needed to address this issue, considering the propensity of these microalgae to form cell clumps, thus preventing adequate exposure of the cells in the middle of clumps to elevated temperatures.

The variable in vitro antimicrobial susceptibility pattern of *Prototheca* strains and species may be one of the causes of antifungal drug treatment failures, and sensitivity of *Prototheca* spp. in vitro does not necessarily correlate with its efficacy in vivo. Therefore, the development of new and effective agents is required for treating human and animal protothecal infections. The prudent use of antibiotics and their replacement with other alternative and preventive measures may potentially further contribute to the control of protothecosis cases.

## Supplementary Materials

The following supporting information can be downloaded at: www.mdpi.com/article/10.3390/microorganisms10050938/s1, Table S1: Detection of *P. zopfii/P. bovis* microalgae in two wastewater metagenomic datasets by bioinformatic methods
