# Supplementary material for: Prototheca Infections and Ecology from a One Health Perspective
Source: Microorganisms. 2022 Apr 29;10(5):938. doi: 10.3390/microorganisms10050938 (PMC9144699; doi:10.3390/microorganisms10050938)
Supplement: Supplementary file 1 [file microorganisms-10-00938-s001.zip › microorganisms-1684651-supplementary.pdf]

Table S1. Detection of *P. zopfii/P. bovis* microalgae in two wastewater metagenomic datasets by bioinformatic methods

Nr.	NCBI Biosample Code	NCBI Biosample	Location	Sample details	NCBI SRA Code	Contig assem-bler	Metagenomic contig identifier	Metagenomic contig length (nt)	Best NCBI Nucleotide collection (nt) database hit		Best NCBI whole-genome shotgun contigs (wgs) database hit	
1.	SAMEA4527652	wastewater metagenome	Karachi, Pakistan	covered sewer line	ERR1713384	MEGAHIT	k141_979044	311	*Prototheca zopfii* var. hydrocarbonea (28S ribosomal RNA gene) FR848898.1	99.36% identity 100% coverage	*Prototheca bovis* strain SAG 2021 Scaffold_21, (28S ribosomal RNA gene) PGFX01001210.1	100% identity 100% coverage
2.	SAMEA4527666	wastewater metagenome	Seattle, USA	wastewater treatment plant	ERR1713398	MEGAHIT	k141_748436	346	*Prototheca zopfii* var. hydrocarbonea (28S ribosomal RNA gene) FR848898.2	99.13% identity 100% coverage	*Prototheca bovis* strain SAG 2021 Scaffold_21, (28S ribosomal RNA gene) PGFX01001210.1	99.13% identity 100% coverage

Nr.	Contig sequence
1.	>k141_979044<br>AGACTACCCGCTGAACTTAAGCATATCAATAAGCGGAGGAAAAGAAACCAACAAGGATTCCCTCAGTAACGGCGAGCGAACCGG<br>GAACAGCCCAGCGTGTCAATCTGGCCTTTCGGGGTCCGAGTTGTGGTCTGGAGGGCGGCGCTCCGCGCGGTCCGATCGCCAAGT<br>CCGCTGGAAAGCGGCGTCGTAGAGGGTGACAACCCCGTGGGCGATCGGCTTCCGCGCTTCTCGAGCCCGTTCCGCGGAGTCGGG<br>TTGCTTGGGAATGCAGCTCTAAGCCGGTGGTAAATCCCATCGAAGGCTAAATATCGGCG
2.	>k141_748436<br>GAAGCGCGGAAGCCGATCGCCCACGGGGTTGTCACCCTCTACGACGCCGCTTTCCAGCGGACTTGGCGATCGGACCGCGCGGAG<br>CGCCGCCCTCCAGACCACAACTCGGACCCCGAAAGGCCAGATTGACACGCTGGGCTGTTCCCGGTTCGCTCGCCGTTACTAAGGG<br>AATCCTGGTTGGTTTCTTTTCCTCCGCTTATTGATATGCTTAAGTTCAGCGGGTAGTCTTGCCTGAGCTCAGGTCCAATGAGAAAGT<br>GAATTCCTGGCATTGGCGACACACGCGAGCCGCGCGCCACCGAGCGACCGCCCGCGAGAGCTGGAGAACGAAGACCCGAGAGT<br>CACCGTC
